# Refining MASLD Phenotypes: Clinical, Metabolic, and Elastographic Differences Between Adipose Tissue Dysfunction and Obesity-Driven Disease

**DOI:** 10.3390/nu17243940

**Published:** 2025-12-17

**Authors:** Tudor Cosma, Lucretia Avram, Valer Donca, Alin Grosu, Laurentiu Stoicescu, Elena Buzdugan, Andrada Nemes, Andrei-Mihai Balan, Dana Crisan

**Affiliations:** 1 Regional Institute of Gastroenterology and Hepatology “Prof. Dr. Octavian Fodor”, Faculty of Medicine, “Iuliu Hatieganu” University of Medicine and Pharmacy, 400012 Cluj-Napoca, Romania; tudorcosma1998@gmail.com; 2 Department 5—Medical Specialties, Clinical Municipal Hospital, Faculty of Medicine, “Iuliu Hatieganu” University of Medicine and Pharmacy, Geriatrics-Gerontology, 400012 Cluj-Napoca, Romania; avram.lucretia9@gmail.com; 3 Department of Internal Medicine, 5th Medical Clinic, Clinical Municipal Hospital, Faculty of Medicine, “Iuliu Hatieganu” University of Medicine and Pharmacy, 400012 Cluj-Napoca, Romania; stoicescul@yahoo.com (L.S.); crisan.dc@gmail.com (D.C.); 4 Intensive Care Unit Department 6, Clinical Municipal Hospital, Faculty of Medicine, “Iuliu Hatieganu” University of Medicine and Pharmacy, 400012 Cluj-Napoca, Romaniabalanandreimihai@yahoo.com (A.-M.B.)

**Keywords:** adipose tissue dysfunction, liver stiffness, obesity, metabolic syndrome

## Abstract

Background/Objectives: Metabolic dysfunction-associated steatotic liver disease (MASLD) is a heterogeneous condition shaped by metabolic dysfunction, adipose tissue distribution, inflammatory activation, and body composition. Understanding how these factors interact across distinct clinical phenotypes is essential for improving diagnostic accuracy and risk stratification. This study aimed to compare metabolic, inflammatory, and elastographic profiles between MASLD subgroups defined by adipose tissue dysfunction (ATD) and obesity, and to identify pathways linking metabolic dysregulation to hepatic fibrosis. Methods: We conducted a cross-sectional observational study including 178 adult participants evaluated clinically, biochemically, and by bioimpedance and shear wave elastography. Participants ranged in age from 19 to 82 years. Patients were stratified into a non-MASLD control group and two MASLD subgroups: MASLD with ATD (G1) and MASLD with obesity (G2). Anthropometric data, lipid profile, glycemic markers, cytokines (IL-6, IL-10, TNF-α), liver stiffness, and non-invasive fibrosis indices were compared across groups using standard statistical testing. Results: Patients with MASLD showed higher liver stiffness, triglycerides, and IL-6/IL-10 levels than controls. Between MASLD phenotypes, the ATD group (G1) exhibited a more inflammatory and dysmetabolic profile, with significantly higher triglycerides, IL-6 levels, neutrophil counts, and creatinine, alongside trends suggesting early sarcopenic changes. In contrast, the obese phenotype (G2) demonstrated greater hepatic structural involvement, including higher liver stiffness and BMI, AST/ALT ratio and Diabetes (BARD) scores, despite more favorable inflammatory parameters. Several associations between liver stiffness, IL-6, and glycemic control approached but did not reach statistical significance. Conclusions: MASLD progression appears to follow two complementary but distinct mechanisms: an inflammatory, adipose dysfunction pathway dominated by IL-6 activation and early anabolic decline, and a metabolic-overload pathway driven by obesity. Phenotype-specific evaluation integrating inflammatory markers, metabolic indices, and elastographic parameters may improve risk stratification and inform personalized therapeutic strategies.

## 1. Introduction

Metabolic dysfunction-associated steatotic liver disease (MASLD), the updated nomenclature for non-alcoholic fatty liver disease (NAFLD), has emerged as the most prevalent chronic liver condition globally, affecting approximately 30–38% of the general population and up to 75% of individuals with obesity or type 2 diabetes mellitus (T2DM) [1,2]. This reconceptualization emphasizes the primacy of metabolic dysfunction over the prior exclusionary definition of NAFLD, anchoring the disease firmly within the broader framework of non-communicable metabolic disorders [1]. MASLD comprises a spectrum of hepatic phenotypes ranging from isolated steatosis to metabolic steatohepatitis (MASH), progressive fibrosis, cirrhosis, and hepatocellular carcinoma [3,4]. Hepatic fibrosis has emerged as the key determinant of liver-related and all-cause mortality in MASLD, surpassing other histopathological features such as steatosis or lobular inflammation [5,6]. The pathogenesis of fibrosis is tightly linked to systemic insulin resistance, chronic low-grade inflammation, oxidative stress, and adipose tissue dysfunction [7,8]. Obesity remains a cardinal risk factor, but increasing evidence underscores the heterogeneous metabolic consequences of different adiposity phenotypes. While metabolically healthy obesity (MHO) may confer relative protection, MASLD itself may hasten metabolic deterioration even in these individuals [9]. In contrast, patients with lipodystrophy—characterized by ectopic lipid redistribution and profound insulin resistance—exhibit a more aggressive disease phenotype [10].

Growing evidence indicates that MASLD encompasses heterogeneous metabolic phenotypes with distinct clinical trajectories. Most cases worldwide are obesity-driven, reflecting the global burden of adiposity and metabolic syndrome, and accounting for an estimated 70–85% of MASLD presentations in population-based cohorts [1,2,3,4]. However, a substantial minority-approximately 10–15% of patients-exhibit disproportionate metabolic impairment relative to BMI, a phenotype attributable to adipose tissue dysfunction (ATD) rather than excessive adiposity [9,11]. ATD-driven MASLD is characterized by impaired adipose storage capacity, ectopic lipid deposition, severe insulin resistance, and heightened inflammatory signaling, features that predispose affected individuals to accelerated hepatic fibrogenesis even in the absence of obesity.

Lipodystrophy, although rare, exemplifies the impact of adipose tissue dysfunction on MASLD severity. Characterized by reduced adipose storage capacity, severe insulin resistance, and ectopic lipid deposition, it is associated with rapid progression to steatohepatitis and fibrosis [7,12]. Metreleptin therapy improves metabolic abnormalities in these patients, but therapeutic aspects are beyond the scope of this study; instead, lipodystrophy serves as a mechanistic model illustrating how impaired adipose function, rather than obesity alone, can drive aggressive MASLD [13].

Chronic subclinical inflammation is a key driver of fibrogenesis in MASLD, mediated by an interplay of cytokines such as IL-6, TNF-α, and IL-1β, which promote hepatocellular injury, immune activation, and hepatic stellate cell (HSC) stimulation [1,7,14]. IL-6 is particularly relevant due to its dual, context-dependent roles: while classic IL-6 signaling may support hepatocyte regeneration, IL-6 trans-signaling via soluble IL-6 receptor (sIL-6R) and glycoprotein 130 (sgp130)-amplifies inflammatory injury and fibrotic remodeling, and higher circulating sIL-6R/sgp130 levels correlate with increased liver stiffness, especially in patients with glucolipotoxic metabolic stress [15,16]. Conversely, IL-6 signaling in myeloid cells can exert anti-fibrotic effects through exosomal release of miR-223, a suppressor of the pro-fibrogenic transcription factor TAZ [17]. This bidirectional behavior reflects the broader complexity of immunometabolic regulation in MASLD. Additional cytokines modulate this environment: TNF-α is often elevated, whereas IL-10 is reduced in advanced disease, especially in individuals with morbid obesity [14,18]. Together, the imbalance of IL-6, TNF-α, and IL-10 sustains a pro-inflammatory hepatic milieu that perpetuates fibrosis progression. Because pro-inflammatory cytokines such as IL-6 and TNF-α promote hepatic stellate cell activation and extracellular matrix deposition, cytokine dysregulation has been consistently associated with increased liver stiffness and higher non-invasive fibrosis scores in MASLD. These mechanistic and clinical correlations provide a strong rationale for integrating elastography into the assessment of inflammatory and structural disease burden.

Genetic predisposition also contributes, with variants in genes such as PNPLA3, TM6SF2, and IL6 associated with enhanced susceptibility to steatosis and fibrosis [15].

The association between MASLD and T2DM is particularly salient. MASLD is both a consequence and predictor of metabolic decompensation, creating a vicious cycle of hepatic and systemic disease progression [4,19]. In T2DM populations, MASLD confers a higher risk of advanced fibrosis, cardiovascular complications, and mortality [19]. Despite its clinical significance, the assessment of hepatic fibrosis still relies heavily on invasive histologic examination. Although non-invasive modalities such as shear wave elastography (SWE), magnetic resonance elastography (MRE), and serologic indices (e.g., FIB-4, APRI, and the Enhanced Liver Fibrosis [ELF] score) are increasingly validated and incorporated into international guidelines, their diagnostic precision remains limited in atypical metabolic phenotypes and early-stage disease [20]. Recent advances have introduced novel biomarkers such as the DA-GAG composite score and AI-enhanced elastographic algorithms, which improve sensitivity and outperform traditional indices in metabolic cohorts [1,20,21]. These developments highlight the need for integrated, multimodal approaches capable of simultaneously assessing inflammatory activity and fibrotic burden in MASLD [14].

Recognizing these phenotype-specific patterns of metabolic dysfunction and fibrotic risk is clinically relevant, as they may inform tailored management strategies-prioritizing anti-inflammatory or insulin-sensitizing approaches in ATD-driven MASLD, while focusing on weight reduction and metabolic load in obesity-driven disease.

The aim of this study was to investigate how adipose tissue dysfunction and obesity influence metabolic, inflammatory, and structural features of MASLD, and to determine their association with liver stiffness and non-invasive fibrosis markers. By comparing distinct MASLD phenotypes, the study sought to identify key pathways linking metabolic dysregulation to hepatic fibrogenesis.

## 2. Materials and Methods

### 2.1. Study Design and Patient Inclusion Criteria

This cross-sectional observational study was carried out at the Municipal Clinical Hospital of Cluj-Napoca, Romania, between May 2023 and March 2024. A total of 178 adult patients aged 21 to 89 years were consecutively enrolled during routine outpatient visits in the Metabolic and Diabetology Departments. Eligible participants were adults with complete anthropometric, laboratory, and imaging data, capable of providing informed consent and completing all study procedures. Patients with acute illness, active infections, inflammatory or decompensated chronic diseases (hepatic, cardiac, or renal failure), excessive alcohol consumption (>20 g/day for women or >30 g/day for men), or known causes of secondary steatosis, such as viral hepatitis or autoimmune liver disease, were excluded. The study was approved by the Ethics Committee of the Municipal Clinical Hospital Cluj-Napoca, and all participants provided written informed consent in accordance with the principles of the Declaration of Helsinki (2013 revision).

No a priori sample size calculation was performed because all consecutive eligible patients were enrolled. A post hoc assessment indicated that the final sample (*n* = 178) provided sufficient power to detect the observed between-group differences, as the magnitude of contrasts in key variables—such as liver stiffness, IL-6, and triglycerides—was consistent with effect sizes typically detectable at >80% power under standard α levels.

### 2.2. Clinical and Laboratory Assessment

Each participant underwent a standardized metabolic and clinical evaluation. Anthropometric measurements included body weight, height, and waist circumference, which were obtained using calibrated instruments. The body mass index (BMI) was calculated as weight in kilograms divided by height in meters squared, and blood pressure was recorded after a 10 min rest using an automated sphygmomanometer. Venous blood samples were obtained after an overnight fast of at least ten hours, between 7:00 and 9:00 AM, to reduce circadian variability in metabolic and inflammatory biomarkers.

**Figure 1:**
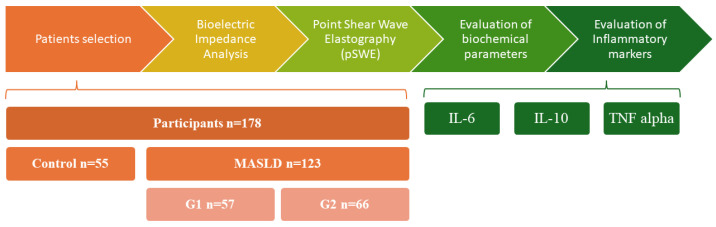
Stages of the observational study. Abbreviations: pSWE—point Shear Wave Elastography; MASLD—Metabolic Dysfunction-Associated Steatotic Liver Disease; IL-6—Interleukin 6; IL-10—Interleukin 10; TNF alpha—Tumor Necrosis Factor alpha.

The laboratory workup comprised hepatic enzymes (ALT, AST, GGT, ALP, and total bilirubin), lipid profile (total cholesterol, HDL-C, LDL-C, and triglycerides), and glycemic indices (fasting plasma glucose and HbA1c). Inflammatory markers IL-6, IL-10, and TNF-α-were quantified using enzyme-linked immunosorbent assay (ELISA) kits, while renal and muscle-related biomarkers, including serum creatinine, cystatin C (Cys-C), and myostatin, were also measured by ELISA. For all biomarkers, we employed commercially available ELISA kits (Orion Biologics, Cluj-Napoca, Romania), including assays for Cystatin C (E-EL-H3643), IL-6 (E-EL-H6156), IL-10 (E-EL-H6154), TNF-α (E-EL-H0109), and Myostatin (EL-H1437), as well as a colorimetric assay for creatinine (E-BC-K188-M). Detailed analytical characteristics provided by the manufacturers—such as detection ranges, assay procedures, and performance specifications—were reviewed to ensure methodological transparency and reproducibility. An overview of the methodological workflow is presented in Figure 1.

### 2.3. Body Composition Analysis

Body composition was analyzed using a multi-frequency segmental bioelectrical impedance analyzer (Tanita MC-780U, Tokyo, Japan). This eight-electrode device estimates segmental fat and lean mass, visceral fat index, phase angle, and basal metabolic rate using alternating electrical currents at multiple frequencies (5–1000 kHz). Measurements were performed in standardized conditions, with participants barefoot, fasting, dressed lightly, and refraining from strenuous physical activity or high fluid intake during the previous 12 h.

### 2.4. Ultrasound and Liver Stiffness Assessment

Hepatic imaging was performed with the ARIETTA 70 ultrasound system (Hitachi, Tokyo, Japan), equipped with real-time shear wave elastography (SWE). The assessment followed the 2024 EASL recommendations for non-invasive fibrosis evaluation. Liver steatosis was graded semi-quantitatively based on parenchymal echogenicity, vascular blurring, and liver-to-kidney contrast, while liver stiffness was measured in the right hepatic lobe through an intercostal approach, with the patient in the supine position and a brief breath-hold. At least ten valid measurements were obtained for each patient, and the median value, expressed in kilopascals (kPa), was recorded for statistical analysis. All SWE examinations were performed by a single experienced investigator who was blinded to the patients’ clinical and laboratory data. Laboratory personnel conducting biomarker analyses were likewise blinded to clinical information.

### 2.5. Patient Stratification

Patients were subsequently classified into three groups according to hepatic imaging and metabolic phenotype. The control group included individuals without hepatic steatosis and without metabolic syndrome. The first MASLD subgroup (Group 1) comprised patients with hepatic steatosis and moderate to severe adipose tissue dysfunction (ATD), defined by the Visceral Adiposity Index (VAI), low BMI, and disproportionate metabolic alterations relative to fat mass. The second MASLD subgroup (Group 2) included patients with hepatic steatosis and obesity (BMI ≥ 30 kg/m^2^) but with absent or mild ATD. The cut-off points of VAI for identification of ATD are shown in Table 1.

VAI was selected because it was the most appropriate index given the biological and anthropometric parameters available in our cohort (BMI, waist circumference, triglycerides, HDL-C), and it is a validated marker of adipose tissue dysfunction in comparable populations [22].

**Table 1:** Age-stratified cut-off points of VAI for identification of adipose tissue dysfunction (ATD). (adapted from [23]).

	ATD Absent	Mild ATD	Moderate ATD	Severe ATD
Age < 30 years	≤2.52	2.53–2.58	2.59–2.73	>2.73
≥30 < 42 years	≤2.23	2.24–2.53	2.54–3.12	>3.12
≥42 < 52 years	≤1.92	1.93–2.16	2.17–2.77	>2.77
≥52 < 66 years	≤1.93	1.94–2.32	2.32–3.25	>3.25
≥66 years	≤2	2.01–2.41	2.42–3.17	>3.17

### 2.6. Statistical Analysis

All statistical analyses were performed using IBM SPSS Statistics version 29.0 (IBM Corp., Armonk, NY, USA). The normality of continuous variables was tested with the Kolmogorov–Smirnov test, and results were expressed as median and interquartile range (IQR). Variables that demonstrated non-normal distributions, were analyzed using non-parametric tests. Group differences were analyzed using one-way ANOVA with Tukey’s post hoc test for parametric data and the Mann–Whitney U test for non-parametric distributions. Categorical variables were compared using the Chi-square or Fisher’s exact test as appropriate. A two-tailed *p* value < 0.05 was considered statistically significant. Missing or incomplete data were excluded from the analysis; no imputation was performed.

## 3. Results

### 3.1. MASLD vs. Non-MASLD: Clinical and Biochemical Profile

The study included 178 participants stratified into three groups: non-MASLD control group (*n* = 55), MASLD with adipose tissue dysfunction (G1, *n* = 57) and MASLD with obesity (G2, *n* = 66). Patients with MASLD were significantly older than controls, with a similar age profile between G1 and G2.

Compared with controls, patients with MASLD exhibited a more adverse metabolic and inflammatory profile, characterized by higher atherogenic lipids, elevated IL-6 and IL-10 concentrations, and increased liver stiffness, while TNF-α levels remained comparable between groups. Non-invasive fibrosis indices, including the FIB-4 score, were also higher among MASLD participants. Full biochemical, inflammatory, and elastographic results are presented in Table 2.

### 3.2. Comparison of MASLD Phenotypes

When comparing the two MASLD phenotypes, several metabolic, fibrotic, and inflammatory differences were observed. Across the two MASLD phenotypes, patients with adipose tissue dysfunction (G1) exhibited a distinctly more dysmetabolic and pro-inflammatory profile, reflected by higher triglycerides, lower HDL, elevated IL-6 concentrations, and increased neutrophil counts. In contrast, the obese phenotype (G2) showed more pronounced structural hepatic involvement, with higher liver stiffness and BARD scores. IL-10 and cystatin C demonstrated numerical but non-significant trends toward higher values in G1, while LDL, TNF-α, and several hematologic indices showed non-significant differences between groups. Full biochemical, inflammatory, and elastographic comparisons are detailed in Table 3.

In summary, patients with MASLD and adipose tissue dysfunction exhibited a more pro-inflammatory and dysmetabolic phenotype, whereas those with obesity showed greater structural hepatic impairment. Although cystatin C showed a non-significant trend toward higher values in G1, this finding should be interpreted cautiously, and further research is required to determine its clinical relevance. These findings delineate two complementary but distinct pathways of liver injury within MASLD: one driven primarily by inflammation and adipose tissue dysfunction, and another dominated by mechanical and metabolic overload associated with obesity.

## 4. Discussion

The complexity of MASLD lies not merely in its phenotypic heterogeneity, but in the dynamic and often paradoxical ways in which metabolic, inflammatory, and fibrogenic pathways interact across distinct patient profiles. Our study, which compared MASLD phenotypes based on clinical, bioimpedance, and elastographic parameters, offers compelling support for the growing consensus that MASLD is not a monolithic condition, but a constellation of overlapping syndromes shaped by adipose tissue distribution, immune signaling, glycemic control, and muscle status.

Our findings revealed two distinct MASLD phenotypes. The adipose-dysfunction group (G1) demonstrated disproportionately increased liver stiffness despite low adiposity, consistent with an inflammation-dominant, IL-6–driven phenotype. In contrast, the obesity-driven group (G2) exhibited classical metabolic overload with moderate fibrosis and a distinct cytokine pattern. These phenotype-specific profiles provide a coherent framework for interpreting the metabolic, inflammatory, and structural differences observed throughout the study.

The most striking and consistently replicated finding is the disproportionately increased liver stiffness observed in the lipodystrophic subgroup (G1), despite the absence of overt obesity or elevated VAI. This pattern aligns with evidence that impaired adipose expandability, rather than total adiposity, drives ectopic lipid deposition and accelerates fibrogenesis in adipose-dysfunction phenotypes [2,9].

By contrast, the MASLD-obese phenotype (G2) demonstrated a more classical metabolic disturbance, with elevated VAI and markers of low-grade inflammation-including neutrophilia, a higher platelet-to-lymphocyte ratio and moderately elevated IL-6 levels-yet with only intermediate fibrosis scores and liver stiffness values. This aligns with data suggesting that a subset of obese individuals, characterized as metabolically healthy obese (MHO), retain a degree of adipose buffering capacity and insulin sensitivity that confers partial protection against hepatic injury [11,24]. Our findings support this paradigm, while also noting that some inflammatory parameters approached statistical significance (e.g., IL-6 with *p* = 0.054), raising the possibility of subclinical fibrotic activation in a subset of MHO patients. These observations reinforce our study aim to differentiate structural versus inflammatory drivers of fibrosis, with liver stiffness acting as a key discriminator between MASLD phenotypes. It is also important to acknowledge the inherent limitations of SWE, including operator dependence, sensitivity to fasting status, and reduced accuracy in individuals with higher BMI, which should be considered when interpreting elastography-based findings.

The obese phenotype also exhibited higher BARD scores compared with the lipodystrophic group, which may be explained by the composite nature of this index: both BMI ≥ 28 kg/m^2^ and the presence of diabetes are scoring components that inherently favor higher results in obese individuals. Therefore, the BARD score in this context likely reflects the algorithmic weighting of metabolic comorbidities rather than a true biological difference in fibrotic activity, consistent with observations from Younossi et al. [8,21].

Although FIB-4 and APRI are widely used non-invasive fibrosis indices, they primarily reflect age-related and hematologic parameters and therefore may lack sensitivity in differentiating early or phenotype-specific fibrosis patterns in MASLD. In contrast, the BARD score, which incorporates BMI and diabetes status, naturally favors higher values in obesity-driven disease, limiting its ability to detect fibrotic risk in ATD-driven phenotypes.

The inflammatory signature proved to be a major differentiator between groups, particularly with regard to IL-6 and IL-10. G2 patients consistently exhibited the highest IL-6 concentrations, which is consistent with recent literature portraying IL-6 as a bifunctional cytokine: regenerative via classical signaling, but proinflammatory and profibrotic via trans-signaling [14]. Moreover, our data revealed elevated IL-10 in G2, a finding that, while seemingly anti-inflammatory, may represent a futile compensatory attempt to counterbalance unresolved cytokine activity—a pattern previously documented in patients with failed anti-fibrotic immune regulation [14,25]. These findings align with established models linking IL-6 dysregulation to metabolically induced fibrogenic signaling, reinforcing its central role in defining the inflammation-dominant profile of ATD-driven MASLD [14,26]. These findings support our initial objective of characterizing ATD-driven MASLD as an inflammation-dominant phenotype, in which IL-6 dysregulation plays a central mechanistic role.

Furthermore, our results contribute to the growing body of evidence emphasizing the synergistic roles of T2DM and sarcopenia in MASLD progression. In our cohort, T2DM was more prevalent in G2 and closely associated with increased liver stiffness and inflammatory markers-consistent with studies identifying T2DM as an independent predictor of advanced fibrosis. Although not all intergroup comparisons reached statistical significance, correlations between glycemic status, IL-6, and elastographic stiffness approached significance, suggesting a latent pathophysiological relationship [27,28].

The interplay between sarcopenia and hepatic fibrogenesis was also evident. Bioimpedance data revealed that G2 patients had lower lean mass and higher extracellular-to-total-body-water ratios, consistent with muscle depletion and subclinical inflammation [3,29,30]. These observations align with cohort studies showing that sarcopenia independently correlates with liver stiffness, even after adjustment for adiposity. In this context, the higher creatinine levels and the upward trend in cystatin C observed in the adipose-dysfunction group suggest early muscle-related alterations [29]. This pattern is consistent with our exploratory objective of assessing whether muscle-related changes might modulate fibrotic risk across MASLD phenotypes.

Another dimension explored is the limited predictive power of classical fibrosis indices in atypical MASLD phenotypes. In G2, FIB-4 and HSI values were frequently in the ‘intermediate’ range despite markedly elevated liver stiffness, echoing concerns that BMI-dependent indices may underestimate fibrosis in individuals with altered body composition, including lean or lipodystrophic patients [3,31]. Novel diagnostic tools—such as cytokine-based panels and AI-enhanced elastography—are, therefore, increasingly advocated for these contexts [32]. Recent evidence also highlights the superior performance of emerging multimarker composites, including the DA-GAG score, which improves the detection of significant fibrosis in MASLD cohorts [33]. Together, these findings underscore the need for integrated, phenotype-aware diagnostic strategies that combine biochemical and imaging-based markers.

Genetic susceptibility may further contribute to phenotype divergence. Variants in PNPLA3, MBOAT7, and IL-6 genes have been shown to modulate lipid partitioning, inflammatory responsiveness, and fibrosis progression [15,34]. Although genotyping was not performed in our cohort, such polymorphisms may partially explain the differential cytokine profiles and fibrotic behavior observed across phenotypes. Therapeutically, these insights are relevant, as IL-6 and IL-11 signaling have emerged as promising targets for metabolic-inflammatory liver disease, with IL-11 shown to potentiate matrix deposition and hepatocyte dysfunction under metabolic stress [35].

Overall, our results reinforce a dual-pathway model of MASLD: one driven primarily by inflammation and adipose-tissue dysfunction, and another dominated by metabolic overload associated with obesity. Recognizing this dichotomy has direct clinical implications, suggesting that anti-inflammatory and insulin-sensitizing therapies may be prioritized for the adipose-dysfunction phenotype, whereas antifibrotic and weight-reduction approaches may be more effective in obesity-driven disease [1].

From a clinical perspective, phenotype-specific assessment has direct therapeutic implications. Patients with ATD-driven MASLD, characterised by disproportionate inflammation and IL-6–mediated signalling, may benefit from strategies targeting inflammatory pathways or insulin resistance, whereas individuals with obesity-driven MASLD may respond more favourably to weight reduction and metabolic load modulation. Non-invasive tools may also require phenotype-aware interpretation: elastography and inflammatory biomarkers may be more informative in ATD phenotypes, while steatosis and metabolic indices may better capture disease burden in obesity-driven MASLD. Integrating these phenotype-specific patterns into routine evaluation could support more tailored risk stratification and therapeutic decision-making

## 5. Study Limitations and Future Perspectives

The present study, despite its strengths in phenotypic stratification and multiparametric assessment, is constrained by several important limitations that warrant acknowledgment. First and foremost, its cross-sectional design precludes definitive causal inferences between metabolic, inflammatory, and structural hepatic alterations. While our results suggest strong associations, particularly between lipodystrophy, elevated IL-6 levels, and liver stiffness, longitudinal studies are essential to validate the directionality and durability of these relationships.

Secondly, the sample size, though sufficient for subgroup comparisons, limited the power to detect significance in several trends that approached conventional statistical thresholds (e.g., IL-6, IL-10, glycemic indices; *p*-values between 0.051 and 0.059). As such, some physiologically plausible associations could not be confirmed at the 95% confidence level, and findings must be interpreted with statistical caution. Several associations that approached statistical significance should be interpreted as exploratory, as the study may have been underpowered to detect small-to-moderate effects despite adequate power for the larger observed contrasts. These trends require confirmation in larger, hypothesis-driven cohorts.

As this was a single-centre study based on patients from one hospital in Romania, the external validity of our findings is limited, and the results may not fully generalise to more diverse MASLD populations. Another important limitation lies in the lack of histopathological confirmation of liver fibrosis. Although we employed shear wave elastography, which is well-validated as a surrogate marker, it does not distinguish between ballooning, lobular inflammation, or fibrosis stage. Similarly, our classification of lipodystrophy relied on clinical and anthropometric criteria, without genetic or imaging confirmation (e.g., DEXA or MRI), leaving room for potential misclassification.

In addition, muscle mass and function were not directly quantified, although indirect indicators such as creatinine, cystatin C, and bioimpedance-derived lean mass suggested possible anabolic impairment. Future studies integrating muscle imaging and functional performance measures could better elucidate the contribution of sarcopenia to MASLD progression. Moreover, although all participants underwent bioimpedance analysis, phase angle-a sensitive marker of cellular integrity and inflammatory burden—was not recorded. Its inclusion could have provided complementary insight into inflammatory and metabolic phenotypes.

Despite these constraints, the consistency of our findings with pathophysiological models and previous clinical cohorts lends robustness to the hypothesis that MASLD heterogeneity demands more than one-size-fits-all diagnostics or therapies. Our study contributes valuable insight into the subtle but impactful divergence between obese and lipodystrophic MASLD phenotypes, highlighting the need for precision hepatology.

## 6. Conclusions

In summary, our findings highlight that hepatic fibrosis in MASLD arises from distinct yet overlapping mechanisms driven by adipose tissue dysfunction, inflammation, and anabolic decline rather than obesity alone. Patients with lipodystrophic features showed greater liver stiffness despite lower fat mass, supporting an IL-6–dominant, sarcopenia-linked pathway of fibrogenesis. Conversely, obese MASLD patients exhibited a milder fibrotic profile but stronger metabolic load. These findings support the integration of phenotype-based assessment into routine MASLD evaluation, with greater attention to inflammatory and sarcopenia-linked pathways in ATD-driven disease and weight-focused strategies in obesity-driven MASLD. Future research should validate phenotype-specific monitoring and therapeutic approaches, particularly interventions targeting IL-6–dominant and muscle-related mechanisms.

## Figures and Tables

**Table 2:** Characteristics of study participants.

	Non-MASLD *n* = 55		MASLD *n* = 123		
Parameter	Median	IQR	Median	IQR	*p* Value
Age (years)	40	26	61	17	<0.001 *
BMI (kg/m^2^)	23.45	4.63	31.27	5.92	<0.001 *
Waist circumference (cm)	79	17	106	14	<0.001 *
SBP (mmHg)	120	10	131	20	<0.001 *
Total adipose tissue (%)	29.60	9.30	30.10	9.10	0.755
Muscle mass (%)	27.20	8.40	29.30	7.40	0.840
Platelets	259	82	248	79	0.207
MPV	10.10	1.30	10.60	1.40	0.011 *
Neutrophils	3.78	1.31	4.13	1.80	0.003 *
Lymphocytes	1.85	0.71	2.04	0.88	0.019 *
NLR	1.87	0.92	1.98	0.91	0.303
PLR	84.55	126.30	118.75	57.17	<0.001 *
Total cholesterol (mg/dL)	183.00	41	202	66	0.003 *
HDL (mg/dL)	48	19	45	10	0.009 *
LDL (mg/dL)	109.20	46.20	127.20	62.60	0.017 *
Triglycerides (mg/dL)	88	47	143	103	<0.001 *
Serum albumin (g/dL)	4.40	0.90	4.60	0.90	0.551
AST (UI/L)	21	9	22	15	0.055
ALT (UI/L)	26	22	25	18	0.121
ALP (UI/L)	71	28	75	27	0.232
GGT (UI/L)	22	16	38	30	<0.001 *
Total bilirubin (mg/dL)	0.60	0.30	0.60	0.50	0.130
Creatinine (mg/dL)	0.73	0.30	0.84	0.26	0.029 *
Left liver lobe (mm)	75	20	85	18	0.003 *
Right liver lobe (mm)	130	25	153	23	<0.001 *
E (kPa)	4.56	1.79	6.29	3.21	<0.001 *
FIB-4	0.67	0.66	1.08	0.71	<0.001 *
APRI	0.22	0.10	0.27	0.18	0.060
BARD	2	1	2	1	<0.001 *
FLI	16	19.08	85.25	23.58	<0.001 *
HSI	23.27	16.09	34.89	7.18	<0.001 *
Triglyceride/Glucose index	3.62	0.30	3.93	0.35	<0.001 *
VAI	1.15	0.88	2.08	1.97	<0.001 *
IL-6 (pg/mL)	30.3	31.86	47.69	67.64	<0.001 *
IL-10 (pg/mL)	1.96	2.04	3.48	2.52	<0.001 *
TNF alpha (pg/mL)	5.25	0.54	5.20	0.64	0.290
CYS-C (ng/mL)	8.20	7.90	7.50	6.20	0.235
	*n*	%	*n*	%	
Women	42	76.4	63	51.2	
Men	13	23.6	60	48.8	

Values are presented as the median and interquartile range (IQR) or percentage (%). Significant differences were estimated by means of the Mann–Whitney U Test. * Differences were considered significant when *p* < 0.05. Abbreviations: MASLD—Metabolic Dysfunction-Associated Steatotic Liver Disease; IQR—Interquartile Range; BMI—Body Mass Index; SBP—Systolic Blood Pressure; MPV—Mean Platelet Volume; NLR—Neutrophil-to-Lymphocyte Ratio; PLR—Platelet-to-Lymphocyte Ratio; HDL—High-Density Lipoprotein; LDL—Low-Density Lipoprotein; AST—aspartate aminotransferase; ALT—alanine aminotransferase; ALP—Alkaline Phosphatase; GGT—gamma-glutamyl transferase; E—Elasticity; FIB-4—Fibrosis 4; APRI—Aspartate Aminotransferase to Platelet Ratio Index; BARD—Body mass index, AST/ALT ratio, and Diabetes; FLI—Fatty Liver Index, HSI—Hepatic Steatosis Index; VAI—Visceral Adiposity Index; IL-6—Interleukin 6; IL-10—Interleukin 10; TNF alpha—Tumor Necrosis Factor alpha, CYS-C—Cystatin C.

**Table 3:** Clinical and biochemical characteristics comparing the studied groups.

	Control (*n* = 55) Non-MASLD		G1 (*n* = 57) MASLD with Adipose Tissue Dysfunction		G2 (*n* = 66) MASLD with Obesity					
Parameter	Median	IQR	Median	IQR	Median	IQR	*p* Value			
							ANOVA All Groups	Post Hoc		
								C–G1	G1–G2	C–G2
Age (years)	40	26	60	17	61.50	17	<0.001 *	<0.001 *	0.999	<0.001 *
BMI (kg/m^2^)	23.45	4.63	31.14	7.58	32.04	5.50	<0.001 *	<0.001 *	0.500	<0.001 *
Waist circumference (cm)	79	17	108	14.50	106	13	<0.001 *	<0.001 *	0.717	<0.001 *
SBP (mmHg)	120	10	130	20	135	26	<0.001 *	<0.001 *	0.090	<0.001 *
Total adipose tissue (%)	29.60	9.30	29.35	8.90	30.90	9.20	0.723	0.835	0.504	0.724
Muscle mass (%)	27.20	8.40	29.60	6.80	28.95	8.40	0.699	0.972	0.829	0.695
Platelets	259	82	252	93	243	73	0.320	1.000	0.402	0.400
MPV	10.10	1.30	10.50	1.40	10.60	1.05	0.017 *	0.088	0.819	0.016 *
Neutrophils	3.78	1.31	4.61	2.26	3.86	1.65	<0.001	<0.001 *	0.002 *	0.290
Lymphocytes	1.85	0.71	2.05	1.06	2.03	0.71	0.022 *	0.016	0.445	0.216
NLR	1.87	0.92	2.02	0.96	1.93	0.87	0.373	0.536	0.376	0.973
PLR	84.55	126.30	118.67	59.11	119.99	51.90	0.001 *	0.004 *	1.000	0.003 *
Total cholesterol (mg/dL)	183.00	41	211	75	198.5	70	<0.001 *	<0.001 *	0.174	0.041 *
HDL (mg/dL)	48	19	42	12	47.50	11	<0.001 *	<0.001 *	<0.001 *	0.852
LDL (mg/dL)	109.20	46.20	114.40	71	128.50	57.95	0.036 *	0.081	0.990	0.049 *
Triglycerides (mg/dL)	88	47	203	56	106.5	42	<0.001 *	<0.001 *	<0.001 *	0.345
Serum albumin (g/dL)	4.40	0.90	4.60	0.95	4.60	0.93	0.517	0.511	0.951	0.674
AST (UI/L)	21	9	22	15	22.50	13	0.150	0.270	0.972	0.162
ALT (UI/L)	26	22	27	19	24.50	19	0.159	0.198	0.987	0.233
ALP (UI/L)	71	28	72	25	77.50	28	0.191	0.914	0.386	0.198
GGT (UI/L)	22	16	43	23	35.50	42	0.014 *	0.546	0.160	0.011 *
Total bilirubin (mg/dL)	0.60	0.30	0.60	0.50	0.60	0.42	0.080	0.406	0.616	0.064
Creatinine (mg/dL)	0.73	0.30	0.85	0.25	0.79	0.23	0.016 *	0.026 *	0.040 *	0.959
Left liver lobe (mm)	75	20	85	17	83	20	0.010 *	0.008	0.524	0.101
Right liver lobe (mm)	130	25	160	20	147	24	<0.001 *	<0.001 *	<0.001 *	<0.001 *
E (kPa)	4.56	1.79	5.55	2.64	6.74	3.43	<0.001 *	0.122	0.019 *	<0.001 *
FIB-4	0.67	0.66	1.04	0.73	1.14	0.72	0.001 *	0.031	0.546	<0.001 *
APRI	0.22	0.10	0.24	0.19	0.29	0.19	0.147	0.586	0.605	0.123
BARD	2	1	2	1	2.50	1	<0.001 *	<0.001 *	0.936	<0.001 *
FLI	16	19.08	86.01	17.75	81.11	27.70	<0.001 *	<0.001 *	0.263	<0.001 *
HSI	23.27	16.09	34.50	8.37	35.43	5.99	<0.001 *	<0.001 *	0.676	<0.001 *
Triglyceride/glucose index	3.62	0.30	4.10	0.27	3.78	0.26	<0.001 *	<0.001 *	<0.001 *	<0.001 *
VAI	1.15	0.88	3.63	1.96	1.59	0.71	<0.001 *	<0.001 *	<0.001 *	0.850
IL-6 (pg/mL)	3	31.86	51.89	86.14	41.69	60.29	<0.001 *	<0.001 *	0.046 *	0.027
IL-10 (pg/mL)	1.96	2.04	3.72	2.46	2.48	2.68	<0.001 *	<0.001 *	0.157	0.083
TNF alpha (pg/mL)	5.25	0.54	5.31	0.62	5.14	0.67	0.109	0.340	0.096	0.816
CYS-C (ng/mL)	8.20	7.90	8.10	6.60	7.05	6.67	0.418	0.758	0.823	0.383
	*n*	%	*n*	%	*n*	%				
Women	42	76.4	28	49.1	35	53				
Men	13	23.6	29	50.9	31	47				

Values are presented as the median and interquartile range (IQR) or percentage (%). Significant differences were estimated by means of using the ANOVA Test with Post Hoc analysis. * Differences were considered significant when *p* < 0.05. Abbreviations: MASLD—Metabolic Dysfunction-Associated Steatotic Liver Disease; IQR—Interquartile Range; BMI—Body Mass Index; SBP—Systolic Blood Pressure; MPV—Mean Platelet Volume; NLR—Neutrophil-to-Lymphocyte Ratio; PLR—Platelet-to-Lymphocyte Ratio; HDL—High-Density Lipoprotein; LDL—Low-Density Lipoprotein; AST—aspartate aminotransferase; ALT—alanine aminotransferase; ALP—Alkaline Phosphatase; GGT—gamma-glutamyl transferase; E—Elasticity; FIB-4—Fibrosis 4; APRI—Aspartate Aminotransferase to Platelet Ratio Index; BARD—Body mass index, AST/ALT ratio, and Diabetes; FLI—Fatty Liver Index, HSI—Hepatic Steatosis Index; VAI—Visceral Adiposity Index; IL-6—Interleukin 6; IL-10—Interleukin 10; TNF alpha—Tumor Necrosis Factor alpha, CYS-C—Cystatin—C.

## Data Availability

The dataset used during the current study is available from the corresponding author upon reasonable request.

## Abbreviations

The following abbreviations are used in this manuscript:

MASLD	Metabolic dysfunction-associated steatotic liver disease
ATD	Adipose Tissue Dysfunction
IL-6	Interleukin 6
IL-10	Interleukin 10
TNF-α	Tumor Necrosis Factor alpha
NAFLD	Non-Alcoholic Fatty Liver Disease
T2DM	Type 2 Diabetes Mellitus
MASH	Metabolic Dysfunction-Associated Steatohepatitis
MHO.	Metabolically Healthy Obesity
IL-1β	Interleukin 1-beta
HSC.	Hepatic Stellate Cell
sIL-6R	Soluble Interleukin-6 Receptor
sgp130	Soluble glycoprotein 130
SWE	Shear Wave Elastography
MRE	Magnetic Resonance Elastography
BMI	Body Mass Index
HbA1c	Glycated Hemoglobin
ELISA	Enzyme-linked immunosorbent assay
Cys-C	Cystatin C
EASL	European Association fot the Study of the Liver
IQR	Interquartile range
SBP	Systolic Blood Pressure
MPV	Mean Platelet Volume
NLR	Neutrophil-to-Lymphocyte Ratio
PLR	Platelet-to-Lymphocyte Ratio
HDL	High-Density Lipoprotein
LDL	Low-Density Lipoprotein
AST	Aspartate aminotransferase
ALT	Alanine aminotransferase
ALP	Alkaline Phosphatase
GGT	Gamma-glutamyl transferase
FIB-4	Fibrosis 4
APRI	Aspartate Aminotransferase to Platelet Ratio Index
FLI	Fatty Liver Index
HSI	Hepatic Steatosis Index
VAI	Visceral Adiposity Index
SNPs	Single-nucleotide polymorphisms
MRI	Magnetic Resonance Imaging
BARD	Body mass index, AST/ALT ratio, and Diabetes
